# Role and Mechanisms of Gut Microbiota in Infectious Diseases: Recent Evidence from Animal Models

**DOI:** 10.3390/biology15030256

**Published:** 2026-01-30

**Authors:** Tao Zeng, Linxue Zuo, Qiaorui Yu, Qiurui Wu, Zhiru Bao, Hairong Xiong, Mei Luo, Bei Li

**Affiliations:** 1 Hubei Key Laboratory of Resource Utilization and Quality Control of Characteristic Crops, College of Life Science and Technology, Hubei Engineering University, Xiaogan 432000, China; ztybdylyh@163.com (T.Z.); 17282327586@163.com (L.Z.); 15872050602@163.com (Q.Y.); 19947629809@163.com (Q.W.); zhirubao@hbeu.edu.cn (Z.B.); xx00114158@hbeu.edu.cn (H.X.); 2 Institute of Resource Biology and Biotechnology, Department of Biotechnology, College of Life Science and Technology, Huazhong University of Science and Technology, Wuhan 430074, China

**Keywords:** gut microbiota, infectious diseases, bacterial infections, viral infection, gut-lung axis

## Abstract

Infectious diseases present persistent and complex challenges to global public health, with conventional antibiotic therapies increasingly limited by antimicrobial resistance, microbiota disruption, and adverse effects. There is a critical need to explore complementary strategies that augment host defense mechanisms without exacerbating these limitations. Accumulating evidence underscores the integral role of the gut microbiota—a diverse microbial ecosystem within the gastrointestinal tract—in regulating systemic immunity and pathogen susceptibility. This review synthesizes recent advances from animal models to delineate the multi-faceted mechanisms by which commensal microbes and their metabolites confer protection against enteric and respiratory infections. Key processes include competitive exclusion for nutrients and ecological niches, production of antimicrobial compounds, reinforcement of intestinal barrier integrity, and orchestration of local and systemic immunity via gut–lung axes. We further discuss the potential of microbiota-targeted interventions to enhance treatment efficacy and patient outcomes. By integrating mechanistic insights with translational applications, this review aims to inform the rational design of next-generation anti-infective strategies grounded in microbial ecology and host immunobiology.

## Simple Summary

Infectious diseases remain a major threat to global health. While antibiotics are essential, they often fail to eliminate pathogens fully and can lead to drug resistance and side effects. This review examines how the gut microbiome—the community of bacteria, viruses, and fungi residing in our digestive system—protects against infections. Evidence based on animal studies, we explain how these microbes fight harmful pathogens by producing antimicrobial substances, competing for nutrients, strengthening the gut barrier, and boosting the body’s immune response—even influencing distant organs like the lungs through the “gut–lung axis”. Understanding these mechanisms paves the way for novel strategies to bolster the body’s natural defenses by targeting modulation of the gut microbiota. This knowledge may help reduce reliance on antibiotics, improve patient outcomes, and offer more sustainable strategies to combat infectious diseases in the future.

## 1. Introduction

Infectious diseases represent a persistent threat to global health, with considerable social and economic consequences. Although in the past few decades, the public health conditions in developed countries have significantly improved, the vaccination rate has continued to increase, and the incidence rate of infectious diseases has been greatly reduced. However, in developing countries, infectious diseases remain one of the main causes of death. For example, the World Health Organization (WHO) estimates that in 2019, there were approximately 10 million cases of tuberculosis (TB) globally, with children under 15 accounting for 12% of new cases and 16% of TB-related deaths [1]. Similarly, gastroenteritis caused by *Salmonella* spp. results in an estimated 93.8 million cases annually, leading to 155,000 deaths worldwide [2]. Influenza, a highly contagious respiratory disease, affects about 1 billion people globally each year, with 3 to 5 million cases of severe illness and 300,000 to 500,000 deaths [3]. The incidence and mortality associated with severe acute respiratory syndrome coronavirus 2 (SARS-CoV-2), the causative agent of coronavirus disease 2019 (COVID-19), have attained unprecedented historic highs. With the initial cases documented in December 2019, COVID-19 exhibited an extremely rapid spread as global confirmed cases exceeded 30 million within just the following 10 months [4]. These statistics underscore the continued importance of infectious disease prevention and management in global public health efforts.

The cornerstone of clinical treatment for infectious diseases remains antibiotics. However, these treatments, while effective in many cases, often fail to fully eradicate pathogens, contributing to the rise of antimicrobial resistance. Additionally, antibiotics can disrupt the host’s microbiota, leading to gastrointestinal disturbance. The overuse and misuse of antibiotics further complicate their effectiveness, emphasizing the need for alternative therapeutic strategies that can reduce reliance on traditional antibiotics, mitigate drug resistance, and preserve the balance of the host’s microbial communities, thereby enhancing treatment efficacy and the quality of life for patients.

The gut microbiota refers to the community of microorganisms, including bacteria, viruses, and fungi, that reside in the digestive tract of humans and animals, primarily in the large and small intestines, forming a vast and complex ecosystem within the host’s gastrointestinal tract. The number of gut microbes is enormous, with approximately 10^14^ in the human body, which is ten times the number of human cells [5], and they account for about 60% of the dry weight of feces. Recent research indicates that the gut microbiota encodes approximately 3.3 million genes, with a total genomic content over 150 times that of the human genome, earning it the moniker of the “second genome” of the human body [6]. The gut microbiota plays a crucial role in the host’s food digestion and nutrient absorption, metabolism, barrier maintenance, immune modulation, and the regulation of the brain–gut axis function. Importantly, dysbiosis in the gut microbiota has been increasingly implicated in the pathogenesis of a wide range of infectious diseases.

In this review, we explored how the microbiota mediates local colonization resistance against enteric invaders and respiratory pathogens. We delved into the intricate relationship between the gut microbiota and infectious diseases, drawing on insights gleaned from animal models to elucidate the mechanisms by which these microbial communities influence disease progression and immune response. We analyzed how microbiota alterations impact pathogen susceptibility and host immunity, providing a foundation for future research and therapeutic strategies.

## 2. Defense Mechanisms of the Gut Microbiota Against Infectious Diseases

### 2.1. Direct Killing or Inhibition of Pathogens’ Growth

**Figure 1:**
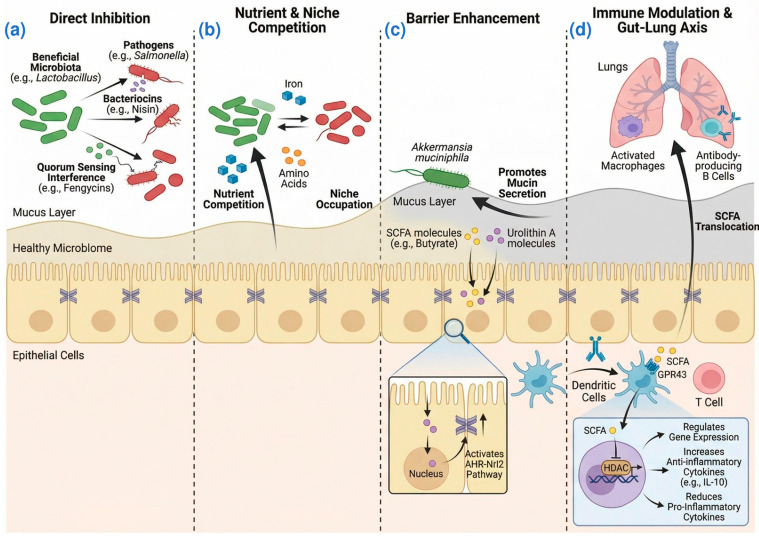
Core Defense Mechanisms of the Gut Microbiota against Infections. Schematic summarizing four key strategies employed by a healthy gut microbiota to confer colonization resistance against pathogens. (**a**) Direct Antagonism. (**b**) Resource Competition. (**c**). Barrier Fortification. (**d**) Immune Priming. Different colors distinguish beneficial microbiota, pathogens, and host cells, while arrows indicate the direction of material exchange and regulatory effects. Created with BioRender.com.

In intricate and overcrowded environments, microorganisms have developed a plethora of defense mechanisms, among which bacteriocins are the most widely distributed. Bacteriocins are short polypeptides produced by bacteria that exhibit antimicrobial activity. For instance, *Enterococcus faecalis* strain expressing bacteriocin 21 has been shown to impede the colonization of other *Enterococcus* strains [7]. Probiotic-derived bacteriocins could assist the host in eliminating pathogenic bacteria. *Lactobacillus salivarius* UCC118 produces the bacteriocin Abp118 and significantly protects mice against *Listeria monocytogenes* infection [8]. Interestingly, the lipopeptide fengycins produced by the probiotic *Bacillus* strain inhibit *Staphylococcus aureus* quorum sensing, which is essential for its intestinal colonization [9]. Another bacteriocin, nisin, which is generated by *Lactococcus* and *Streptococcus* species, has demonstrated efficacy against both Gram-positive and Gram-negative pathogens such as *Clostridioides difficile* [10] and *S. aureus* [11]. In recent years, novel nisin variants have been bioengineered by researchers and have shown great therapeutic potential for treating human diseases [12]. The mechanism of direct pathogen inhibition by gut commensals is illustrated in Figure 1a.

### 2.2. Nutrient Competition and Ecological Niches Occupation

The gut microbiota competes with invading pathogens for limited nutrients such as iron, vitamins, and amino acids. By efficiently occupying key ecological niches and consuming essential nutrients, the resident microbes limit the growth and survival of pathogens. *Escherichia coli* Nissle 1917, a probiotic strain, can outcompete *Salmonella* Typhimurium for iron, thereby reducing its colonization in mouse models of intestinal inflammation [13]. Intraspecific competition for nutrients is more pronounced because they share similar metabolic requirements, leading to a higher likelihood of occupying the same niche within the intestinal tract. Competition for proline with indigenous *E. coli* strains in gnotobiotic mice plays a significant role in the elimination of *E. coli* O157:H7 from the intestine [14]. Primary bile acids synthesized by the host liver are converted into secondary bile acids, such as deoxycholic acid and lithocholic acid, through the action of gut microbiota [15]. Healthy gut microbiota promotes colonization resistance against *C. difficile* by metabolizing bile acids to reduce primary bile acids that prompt spore germination and increase secondary bile acids (SBAs) that impede vegetative growth [16]. Additionally, apart from nutrient competition, invading pathogens also have to compete for physical space with indigenous bacteria. *Bacteroidetes* are the pioneers in the gut, settling in the intestine from infancy. The stable colonization of germfree mice with *Bacteroides. fragilis* prevents subsequent colonization by its isogenic sister cells, indicating that the initial *B. fragilis* population has filled all available niches and is resistant to random displacement [17]. This competition for nutrients and space is illustrated in Figure 1b.

### 2.3. Maintenance and Enhancement of Intestinal Barrier

The gut barrier is a multifaceted structure comprising the inner and outer mucus layers, the epithelial barrier, and the associated immune barrier. The mucus layer serves as the initial physical barrier in the gut, preventing direct bacterial contact with epithelial cells [18]. Gut microbiota could help to maintain the integrity of the intestinal mucosa and enhance mucosal barrier function. *Akkermansia* is widely accepted as the new generation of probiotics [19]. *Akkermansia*-derived mucolytic enzymes can help the host renew intestinal epithelial cells (IECs) and maintain the intestinal barrier [20]. Another study showed that a Western-style diet in mice alters microbiota composition, leading to increased mucus penetrability [21]. Administration of *Bifidobacterium longum* restores mucus growth in these mice, suggesting that distinct bacteria are crucial for gut barrier function [21]. Some microbial metabolites are also found to be important players in this context. In the large intestine, the gut microbiota generates short-chain fatty acids (SCFAs) through fermentation of undigested dietary fibers, mainly including acetate, propionate, and butyrate [22]. SCFAs provide a major energy source for colon epithelial cells [23] and are involved in maintaining the intestinal epithelial barrier [24]. Another microbial metabolite, urolithin A, could enhance gut barrier function and reduce inflammation through the activation of aryl hydrocarbon receptor–nuclear factor erythroid 2-related factor 2 (AhR-Nrf2) pathways, upregulating tight junction proteins and attenuating colitis in mouse models [25]. The role of the gut microbiota in reinforcing the intestinal barrier is outlined in Figure 1c.

### 2.4. Modulation of Host Immune Response

Early-life colonization with microbiota is essential for the adequate development of the immune system. Gut microbiota is involved in the maturation and differentiation of B cells in the neonatal immune system [26]. In addition, due to the presence of a large number of immune cells in the intestinal mucosa, gut microbiota could regulate the activity of the host immune system through interactions with these immune cells. *Bacillus subtilis* potentially mitigates intestinal inflammation by suppressing nuclear factor-κB (NF-κB) signaling in IECs and reducing the upregulation of inducible Nitric Oxide synthase (iNOS) protein levels [27]. It has been reported that a *Lactobacillus gasseri* strain blocked the production of the proinflammatory cytokines tumor necrosis factor (TNF) and interleukin-6 (IL-6) in *Helicobacter pylori* infection by reducing the expression of a disintegrin and metalloprotease 17 (ADAM17) [28].

Metabolites output by intestinal bacteria are critical for host–microbiota interactions and stimulate host immune responses. SCFAs, especially butyrate, have beneficial effects on the host immune system [29,30]. Our previous research has shown that *Saccharomyces boulardii*-derived polysaccharides and polypeptides increased the levels of the microbial metabolite SCFAs, thereby alleviating intestinal barrier dysfunction and inflammation in a human colonic microbiota model [31]. G protein-coupled receptor (GPCR) activation and histone deacetylase (HDAC) inhibition are regarded as the principal mechanisms through which SCFAs modulate host immune function. Butyrate can activate G protein-coupled receptor 109A (GPR109A) to stimulate macrophages and dendritic cells, regulate Treg cell differentiation, while HDAC3 inhibition by butyrate induces macrophage differentiation and augments their antimicrobial defense function [32,33]. The aryl hydrocarbon receptor (AhR), a ligand-dependent transcription factor widely expressed in both immune and non-immune cells of the gut, plays a pivotal role in modulating immune cell development, differentiation, and secretion functions. AhR ligands produced by gut microbes modulate intestinal immune homeostasis through the activation of the AhR [34]. A recent elegant study has delineated the inhibitory effects of tryptophan-derived metabolites from gut microbiota on pathogenic bacteria, revealing specific mechanisms [35]. The microbial metabolites indole-3-ethanol (IEt), indole-3-pyruvic acid (IPyA), and indole-3-aldehyde (I3A), acting as ligands for the dopamine receptor D2 (DRD2), activate DRD2 expression and the GTP-binding protein βγ subunit–phospholipase C–protein kinase Cθ (Gβγ-PLC-PKCθ) signaling pathway in IEt, promoting the degradation of neural Wiskott–Aldrich syndrome protein (N-WASP), a protein required for *Citrobacter rodentium* adhesion to IECs [35].

A healthy gut microbiota is not only pivotal for the intestinal immune system, but its immunomodulatory effects on respiratory infectious diseases have also garnered increasing recognition. Orally administered *Clostridioides butyricum* enhances resistance to influenza virus infection by promoting interferon-λ production in lung epithelial cells through GPR120 and interferon regulatory factor (IRF)-1/-7 activations [36]. In addition, the gut–lung axis, which represents the bidirectional communication pathway between the gut microbiota and the lungs, is gradually being discovered. The mesenteric lymphatic enables bacterial metabolites (such as SCFAs) to cross the intestinal barrier, enter the systemic circulation, and regulate the lung immune response. Gut microbiota-derived SCFAs can be transported to the lung and reduce inflammation through both T cell- and dendritic cell (DC)-dependent mechanisms [37]. SCFAs enhance cluster of differentiation (CD)8^+^ T cell effector functions, establishing immune balance and facilitating resolution of influenza infection [38]. The immunomodulatory mechanisms, including the gut–lung axis, are presented in Figure 1d.

## 3. The Role and Mechanisms of Gut Microbiota in Infectious Diseases

### 3.1. Gut Microbiota and Salmonella Typhimurium Infection

The gut microbiota plays a critical role in modulating host susceptibility to *S. typhimurium*, a major zoonotic pathogen. The use of enrofloxacin, an antibiotic frequently administered in poultry, alters the gut microbiota composition, increasing the abundance of certain bacterial genera such as *Coprococcus*, and diminishing the colonization resistance to *S. typhimurium* infection in chickens [39]. This reveals the potential side effects of antibiotics and highlights the delicate balance between gut microbiota and *S. typhimurium* colonization.

Commensal *E. coli* provides colonization resistance against *S. typhimurium* through mechanisms such as competitive exclusion. Research showed that microbiota-directed bacteriophages targeting *E. coli* or *E. faecalis* in a gnotobiotic mouse model increased susceptibility to *S. typhimurium* [40]. Specifically, neutrophil recruitment during intestinal inflammation primes the elimination of *Salmonella* by commensal *E. coli* through context-dependent mechanisms that enhance bacterial competition [41]. Additional studies have identified specific probiotic interventions that can enhance resistance to *S. typhimurium*. *Akkermansia muciniphila* was found to reduce host susceptibility to *S. typhimurium* infection in mice by promoting NOD-like receptor protein 3 (NLRP3) inflammasome activation and enhancing macrophage antimicrobial functions [42]. A recent study showed that *Enterocloster clostridioformis* protects against *Salmonella* pathogenesis by enhancing epithelial barrier function and modulating mucosal immune responses, including increasing the expression of the host defense peptide resistin-like molecule β (Retnlb/RELMβ) and increasing the abundance of CD4^+^ T-regulatory cells (Tregs) [43]. Concurrently, *Lactobacillus acidophilus* alleviates *Salmonella*-induced inflammatory and oxidative responses in mice through activation of the p62-Keap1-Nrf2 signaling pathway and modulation of cecal microbiota composition [44]. Additionally, *Terminalia bellirica* extract was also found to be able to modulate the gut microbiota composition, increasing the abundance of *Firmicutes* and *Deferribacteres*, *Ruminococcus*, and *Oscillospira* at the genus level, and preventing *Salmonella* infection in mice [45]. Another study demonstrated that specific amino acids such as arginine and tryptophan regulate *Salmonella* colonization through microbiota-dependent mechanisms, limiting pathogen access to these essential nutrients [46].

**Figure 2:**
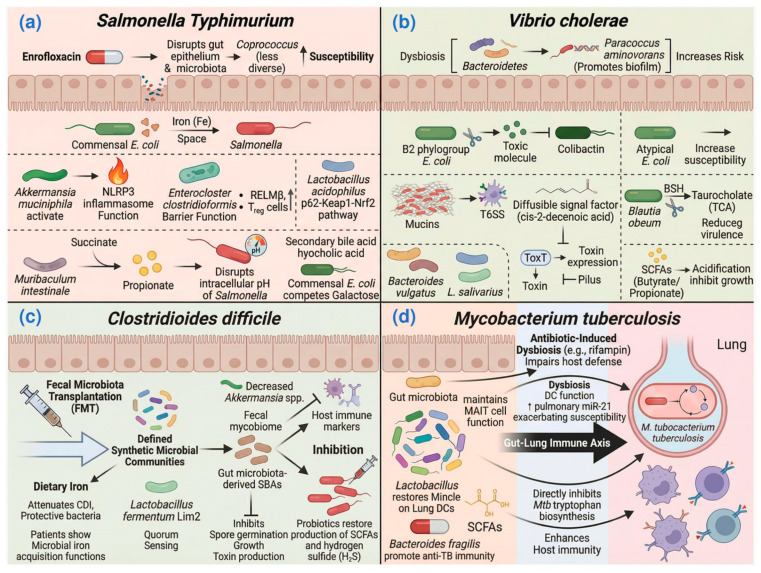
Gut Microbiota Mechanisms in Bacterial Infections. (**a**) *S. typhimurium* infection. (**b**) *V. cholerae* infection. (**c**) *C. difficile* infection. (**d**) *M. tuberculosis* infection. Different colors distinguish beneficial microbiota, pathogens, and host cells, while arrows indicate the direction of material exchange and regulatory effects. Created with BioRender.com.

The mechanisms of probiotics against *S. typhimurium* are closely related to their metabolites. Recent studies have elucidated complementary microbial defense strategies. The gut microbiota-derived secondary bile acid hyocholic acid enhances host defense against *Salmonella* in neonatal rats by potentiating type 3 immunity [47]. Azcarate-Peril and Cui also provided insights into how manipulations of the gut microbiota through prebiotic galacto-oligosaccharides and protocatechuic acid, respectively, can improve resistance to *Salmonella* infection in chickens [48,49]. Propionate, one of the major SCFAs produced by gut microbes, was demonstrated to have the ability to directly inhibit *S. typhimurium* growth by disrupting intracellular pH homeostasis [50]. Notably, *Muribaculum intestinale* specifically restricts *Salmonella* colonization through its metabolic capability to convert succinate to propionate [51]. Interestingly, Shelton et al. found that *S.* Typhimurium can overcome the inhibitory effects of propionate by using it as a carbon source for anaerobic respiration [52]. Furthermore, in aged hosts, melatonin treatment confers protection against multidrug-resistant *Salmonella* infections by promoting microbiota-derived butyrate production, which enhances host defense through immunomodulatory effects [53]. However, strain-specific galactose utilization by commensal *E. coli* creates nutritional competition that limits this carbon source availability for *Salmonella*, thereby mitigating its gut establishment [54]. This mutual antagonism between the indigenous gut microbiota and pathogenic bacteria reveals a complex evolutionary relationship (Figure 2a).

### 3.2. Gut Microbiota and Cholera

Cholera, a severe diarrheal disease caused by *Vibrio cholerae*, remains a significant global health threat, particularly in regions with inadequate sanitation and access to clean water. The gut microbiota also plays a central role in shaping the colonization dynamics of *V. cholerae*, the causative agent of cholera. The indigenous gut microbiota helps prevent *V. cholerae* invasion through a variety of mechanisms, including nutrient competition, antimicrobial peptide production, and the maintenance of the intestinal barrier. Transplanting the human gut microbiome into germ-free and antibiotic-treated mice showcased the impact of variations in the gut microbiome on *V.* colonization [55]. Dysbiosis, marked by an imbalance in microbial communities, has been linked to increased susceptibility to *V. cholerae* infection. Recent studies have elucidated the contributions of specific microbial strains and their metabolites to *V. cholerae* colonization. *Paracoccus aminovorans* promotes *V. cholerae* colonization through biofilm formation, demonstrating the importance of *P. aminovorans* strain in *V. cholerae* infection [56]. Different strains within the same genus may have completely different impacts on the colonization of *V. cholerae*. For example, an atypical *E. coli* strain was identified that increased susceptibility to *V. cholerae* infection [57] while a B2 phylogroup *E. coli* strain was found particularly effective in inhibiting *V. cholerae* growth due to its production of the genotoxin colibactin [58]. This complexity highlights the importance of detailed strain-level analysis in understanding and combating infectious disease.

Further investigations into microbial metabolites have also unveiled their potential role in modulating *V. cholerae* infectivity (Figure 2b). Mucins, which are produced by the gut microbiota, could activate the type VI secretion system (T6SS), a critical virulence factor of *V. cholerae* [59]. In contrast, the diffusible signal factor (DSF), particularly cis-2-decenoic acid produced by commensal Proteobacteria such as *Enterobacter sakazakii*, binds directly to the virulence regulator ToxT in *V. cholerae*. This interaction blocks ToxT binding to toxin gene promoters and accelerates ToxT degradation, thereby potently inhibiting cholera toxin and toxin co-regulated pilus expression [60]. Additionally, the bile salt hydrolases produced by *Blautia obeum* degrade taurocholate, a bile salt that promotes virulence gene expression in *V. cholerae* [55]. Gut microbiota-derived indole disrupts interspecies communication between *V. cholerae* and enteropathogenic *E. coli*, influencing pathogen virulence [61]. These findings suggest that bile acids and other microbial metabolites can influence the pathogenicity of *V. cholerae*. SCFAs have also been implicated in conferring resistance to *V. cholerae*. *Bacteroides vulgatus* produces butyrate and propionate help the host to prevent *V. cholerae* colonization [62]. Similarly, SCFAs from *L. salivarius* LS-ARS2 exert direct antibacterial effects by acidifying the environment and disrupting intracellular pH balance in *V. cholerae* [63]. Furthermore, prebiotic interventions that enhance SCFA production have been shown to improve the efficacy of oral cholera toxin vaccines [64], suggesting that modulating the microbiota may offer novel therapeutic strategies against cholera.

### 3.3. Gut Microbiota and Clostridioides Difficile Infection

*C. difficile*, a spore-forming Gram-positive anaerobic bacterium, has emerged as a major intestinal pathogen globally. Recent studies have increasingly highlighted the effect of the gut microbiota in the pathogenesis and treatment of *C. difficile* infection (CDI). Recently, a designed synthetic microbiota of 37 strains was shown to suppress *C. difficile* in mice, with mechanistic studies revealing that competitive nutrient utilization via Stickland fermentation by a single strain was both necessary and sufficient for this protection [65].

The interplay between iron metabolism and the gut microbiota represents a significant factor in *C*. *difficile* infection. Dietary iron was shown to attenuate CDI, with studies indicating modulation of the intestinal immune response and the enrichment of specific protective bacteria such as *Escherichia coli* AVS0501 [66]. These collective findings underscore that both host iron intake and the iron-related functional capacity of the gut microbiota are associated with the state of CDI. In addition to these iron-related effects, other specific microbial functions have been identified as protective against CDI. *Lactobacillus fermentum* Lim2, isolated from kimchi, could inhibit *C. difficile* toxin production by disrupting its quorum-sensing system [67]. Notably, gut microbe-derived SBAs inhibit *C. difficile* spore germination, growth, and toxin production, highlighting their role in gut microbial resistance to *C. difficile* [16]. Additionally, *C. difficile* induces the production of indole by the gut microbiota, impairing microbial recovery following antibiotic-induced dysbiosis [68]. Probiotics may also help restore the production of SCFAs and hydrogen sulfide (H_2_S), metabolic products that are often diminished during infection [69]. These findings emphasize the therapeutic potential of specific microbial metabolites in preventing and treating CDI. Continued research into the safety, efficacy, and optimal delivery of such interventions is critical for advancing therapeutic strategies (Figure 2c).

### 3.4. Gut Microbiota and Tuberculosis (TB)

TB, an ancient scourge caused by *Mycobacterium tuberculosis* (Mtb), continues to be a major global health challenge, with new diagnostic and treatment strategies being crucial for controlling the disease. While Mtb can cause both pulmonary and extrapulmonary disease, this section focuses on pulmonary TB. In recent years, the profound impact of antibiotics on the gut microbiome and its subsequent influence on TB has garnered significant attention. Studies have shown that antibiotic-induced dysbiosis can persist long after TB therapy, impacting microbial diversity and immune function. There are long-lasting gut microbiota alterations following TB treatment, which could potentially influence the progression of TB and response to therapy [70]. Dysbiosis resulting from rifampin treatment also impairs host defenses against Mtb [71].

Recent findings indicate the effect of the gut microbiota on modulating lung immunity (Figure 2d). Gut microbiota was found to play a key role in early defense against lung colonization by Mtb, potentially through maintaining mucosal-associated invariant T (MAIT) cell function [72]. Gut dysbiosis impairs the function of dendritic cells (DCs), which are crucial for initiating immune responses to Mtb [73]. In another study, it was found that gut microbiota depletion exacerbates TB susceptibility by increasing pulmonary miR-21, impairing anti-TB immunity [74].

Administration of certain probiotics, such as *Lactobacillus,* restored mincle expression on lung DCs along with anti-Mtb response [73]. The underlying mechanisms of these probiotics against Mtb have been increasingly reported. The strain *B*. *fragilis* was identified as a direct regulator of lncRNA promoting anti-TB immunity through lncRNA regulation [75]. In addition, metabolites produced by the gut microbiota also play a role in TB. A prominent example is indole-3-propionic acid (IPA), a gut microbiota-derived tryptophan metabolite, which exerts antitubercular effects through dual mechanisms: directly inhibiting Mtb tryptophan biosynthesis to deprive the pathogen of this essential amino acid, and indirectly enhancing host immune defenses via immunomodulation [76,77]. Beyond tryptophan derivatives, other microbially produced metabolites are also implicated in host defense. Gut microbiota-derived SCFAs such as acetate and butyrate have been identified as critical modulators of antiviral immunity. Intestinal SCFAs are reported to have the ability to transport to the lung [37] and may recast the lung microbiome and immunity via the “gut–lung axis” in TB [78]. These active substances in the host intestine can affect the occurrence and progression of TB through the “gut–lung axis”, offering promising avenues for probiotic-based interventions in TB treatment.

### 3.5. Gut Microbiota and Influenza Infection

Influenza is a highly contagious respiratory illness caused by the influenza virus, which poses a significant seasonal threat to public health, particularly among vulnerable populations. A growing body of evidence highlights the gut microbiota as a key regulator of host defense. Changes in gut microbial composition, such as the expansion of *Bifidobacterium animalis* and *A. muciniphila*, have been shown to enhance host defense against influenza in animal models [79,80,81]. Mechanistically, several gut microbes, including *Clostridioides* sp., *Phocaeicola sartorii*, and *A. muciniphila*, can upregulate the level of N-acetyl-D-glucosamine (GlcNAc) in the mouse gut, which mediates the anti-influenza effect by increasing the proportion and activity of natural killer (NK) cells [79]. Recently, an impressive study has demonstrated that segmented filamentous bacteria (SFB), another key component of the gut microbiota, enhanced antiviral immunity through the activation of alveolar macrophages [82], providing further evidence of the gut–lung axis in viral infections.

**Figure 3:**
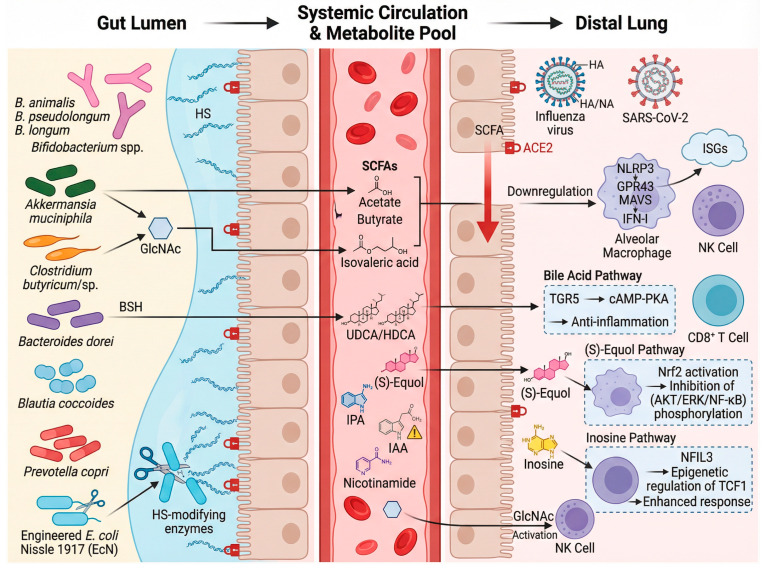
Gut Microbiota in Viral Respiratory Infections. Gut–lung axis communication primes antiviral immunity and resolves inflammation. Different colors distinguish beneficial microbiota, pathogens, and host cells, while arrows indicate the direction of material exchange and regulatory effects. Created with BioRender.com.

The protective effects of gut microbes are largely mediated by their bioactive metabolites. The most frequently reported gut microbial metabolites that can influence influenza are SCFAs (Figure 3). Acetate, produced by *Bifidobacterium pseudolongum*, augments type I interferon (IFN-I) production via the NOD-, LRR- and pyrin domain-containing 3–GPR43–mitochondrial antiviral signaling protein (NLRP3-GPR43-MAVS) signaling axis, safeguarding mice against influenza infection [83]. Notably, acetate’s antiviral role is compromised in high-fat diet settings where its production is reduced, leading to worsened infection outcomes [84]. Additionally, Qiu’s study showed that acetate-mediated metabolic reprogramming optimized virus-specific CD8^+^ T cell responses, as demonstrated by the protective effect of *Blautia coccoides* in mice [85]. The pioneering work of Saint-Martin et al. reported that butyrate, another SCFA, regulated interferon-stimulated genes (ISGs) in chicken respiratory epithelial cells, highlighting its role in shaping antiviral immunity along the gut–lung axis [86]. Beyond SCFAs, a diverse array of microbial metabolites contributes to antiviral defense. Recently, a study demonstrated that the metabolite inosine, derived from gut commensals like *Bifidobacterium*, is crucial for programming protective CD8^+^ T cell immunity against influenza in early life by enabling nuclear factor, interleukin 3 regulated (NFIL3)-dependent epigenetic regulation of T cell factor 1 (TCF1) [87]. Secondary bile acids, such as ursodeoxycholic acid (UDCA) and hyodeoxycholic acid (HDCA), which are restored by *Bacteroides dorei*, activate the lung Takeda G protein-coupled receptor 5 (TGR5)–cyclic adenosine monophosphate (cAMP)–protein kinase A (PKA) pathway to suppress inflammation during influenza infection [88]. Similarly, the gut-derived metabolite (S)-Equol alleviates viral pneumonia by activating the transcription factor Nrf2 in macrophages, thereby inhibiting the pro-inflammatory protein kinase B (AKT)/extracellular regulated protein kinase (ERK)/nuclear factor-kappa B (NF-κB) signaling cascades [89]. The tryptophan metabolism pathway also yields influential immunomodulators. A recent study underscores the significance of the gut microbiota-derived tryptophan metabolite IPA, demonstrating that antibiotic treatment targeting IPA-producing bacteria in influenza-infected mice increases viral load and pulmonary inflammation, while IPA supplementation mitigates these effects [90]. However, the effects of tryptophan metabolites can be complex and context-dependent, as illustrated by indole-3-acetic acid (IAA), which has been associated with aggravated lung injury in bacterial pneumonia models through the AhR [91]. These findings not only deepen our understanding of the gut–lung axis in viral infections but also offer potential therapeutic targets for mitigating influenza severity through microbiota modulation.

### 3.6. Gut Microbiota and COVID-19

SARS-CoV-2 is a highly contagious and pathogenic virus that emerged in 2019. It has led to the COVID-19 pandemic, posing a significant threat to global health and safety. A key insight from the pandemic is the profound influence of the gut microbiota on both disease severity and immune response outcomes in COVID-19.

The mechanisms by which the gut microbiota affects COVID-19 severity are multifaceted (Figure 3). From a functional perspective, these mechanisms operate at multiple levels. Beyond compositional shifts, functional interactions are key. Certain gut commensals can directly modulate host susceptibility by grooming the intestinal glycocalyx; bacteria expressing heparan sulfate-modifying enzymes reduce SARS-CoV-2 spike protein binding and cellular infectivity, a mechanism that has been leveraged by engineering the probiotic *Escherichia coli* Nissle 1917 [92]. At the molecular level, microbial metabolites play a central role. Brown’s cutting-edge study showed that SCFAs downregulated the expression of the SARS-CoV-2 entry receptor angiotensin-converting enzyme 2 (ACE2) [93]. This reduction in ACE2 expression may help mitigate viral entry and, consequently, lower viral load. Additionally, SCFAs activate GPR41 and GPR43, which are involved in enhancing antiviral immunity [93]. Subsequently, Song et al. found that dietary interventions, such as the supplementation of prebiotics like inulin, ameliorated SARS-CoV-2 infection severity by promoting beneficial microbial populations and enhancing SCFA production [94]. The importance of microbial metabolism is further highlighted by interventional studies. In a randomized controlled trial, supplementation with nicotinamide, a modulator of tryptophan metabolism, accelerated recovery in patients with mild-to-moderate COVID-19, an effect paralleled by modulation of gut metagenomic signatures [95]. Together, these insights underscore that further research is essential to develop targeted therapies that leverage the gut microbiota to improve both infection management and immune response.

## 4. Discussion

### 4.1. Limitations and Complementary Value of Preclinical Model Systems

The insights presented in this review are predominantly derived from rodent models, notably germ-free (GF) and specific pathogen-free (SPF) mice, which have been foundational for establishing causal links between the gut microbiota and host defense. However, a direct translation of these findings to human physiology necessitates a critical appraisal of each model’s inherent constraints.

A primary limitation stems from the simplified, low-diversity microbial communities in GF/SPF mice, which represent a drastic reduction in the complex human gut ecosystem. This simplification can lead to an overestimation of the effect size attributable to a single microbial strain or metabolite. In humans, such effects may be attenuated due to functional redundancy, metabolic conversion, and niche competition within a richer community. Complementary insights are gained from poultry models, which offer a pathologically relevant context for specific enteric pathogens like *S. typhimurium*, elucidating how farm-level practices (e.g., enrofloxacin use [39]) or dietary supplements (e.g., protocatechuic acid [49]) modulate colonization resistance. While invaluable for understanding infection dynamics in a key reservoir, the phylogenetic distance from humans limits direct mechanistic extrapolation.

Furthermore, common experimental interventions like broad-spectrum antibiotic depletion introduce significant confounders. Antibiotics can directly induce intestinal epithelial stress and systemic immunomodulation, thereby complicating the interpretation of post-treatment phenotypes and making it difficult to attribute such outcomes solely to “microbiota absence”. The humanized mouse model, colonized with human fecal microbiota or defined synthetic consortia [65], serves as a crucial intermediary. However, it remains imperfect: the engrafted human microbes operate within a murine immune context that may not fully recapitulate human-specific crosstalk, and challenges with stable engraftment and donor variability persist. Porcine models, with their striking physiological and anatomical similarities to humans, address some of these limitations and represent a highly relevant, albeit logistically demanding, system for translational research.

Given these interspecific differences, the findings from animal models must be interpreted with caution, as they can overstate the microbiota’s impact on human disease [96]. This underscores the irreplaceable role of clinical and observational human studies in validating ecological principles. Longitudinal patient analyses have been pivotal, directly linking specific gut microbiota configurations and functional capacities (e.g., iron acquisition genes) to *C. difficile* infection states [97] and connecting microbiome shifts during tuberculosis therapy to inflammatory outcomes [98]. Critically, these clinical insights are now being translated into tangible therapeutic strategies. This translation is exemplified by the recent U.S. Food and Drug Administration (FDA) approval of SER-109, an orally administered live biotherapeutic composed of purified Firmicutes spores for preventing recurrent CDI [99], alongside positive Phase II clinical trial results for the defined bacterial consortium VE303 [100]. These studies illustrate the potential of microbiota-based interventions.

Looking forward, the integration of advanced ex vivo systems, such as human organoids, with emerging technologies like single-cell RNA sequencing, promises to yield more physiologically relevant platforms for mechanistic discovery and therapeutic screening [101,102,103], ultimately bridging the translational gap between preclinical models and human application.

### 4.2. Integrated Ecosystem Dynamics of the Gut Microbiota

While the historical focus on linear, single-factor mechanisms has provided foundational insights, the true nature of colonization resistance demands a shift towards an ecological framework. It is an emergent property of the gut microbiota as a complex adaptive system, arising from community-level interactions characterized by cooperation, competition, and co-evolution with the host [104]. Core principles such as functional redundancy, obligate cross-feeding, and collective niche occupation collectively orchestrate this defense [105]. Colonization resistance relies on a stable, healthy intestinal ecosystem characterized by a highly diverse commensal microbiota, controlled immune homeostasis, and intricate metabolic networks, whereas intestinal pathogens and antibiotic perturbations disrupt this harmony to subvert host defenses.

The most compelling validation of this ecosystem-level logic comes from the rational design of synthetic microbial consortia (SynComs). For instance, foundational work demonstrated that protection against *C*. *difficile* by a defined 37-strain community was achieved not via a single antimicrobial, but specifically through one member’s Stickland fermentation activity, which mediated community-level suppression via targeted nutrient competition [65]. In poultry, a 10-strain SynCom was shown to enhance resistance to *Salmonella* by promoting the establishment of segmented filamentous bacteria, thereby driving a protective Th17 immune response in the host [106]. Similarly, in the context of colorectal cancer associated with *Fusobacterium nucleatum*, a 7-strain SynCom designed using machine learning and metabolic network analysis was able to inhibit the pathogen, alleviate inflammation, and correct metabolic dysregulation, showcasing the predictive power of ecological modeling for therapeutic design [107]. Together, these studies underscore that effective microbiome-based interventions can be achieved through the rational assembly of microbial guilds based on a deep understanding of their functional interaction.

Recent research has unveiled a dynamic, tripartite dialogue within the gut ecosystem, where the host is an active architect rather than a passive vessel. A study demonstrated that the host protein apolipoprotein L9 (APOL9) selectively binds to *Bacteroidales* via a specific lipid (Ceramide-1-phosphate, Cer1P), inducing them to release immunomodulatory vesicles that train intestinal T cells, exemplifying precise host-guided management of commensals [108]. Beyond dedicated immune proteins, the host also exerts control through physiological signals; for instance, host-secreted digestive enzymes can reprogram the metabolism of commensals like *Lactiplantibacillus plantarum* to boost anti-pathogen metabolite production [109]. Pathogens, however, deploy sophisticated ecological counter-strategies. Facing this host–commensal alliance, *S. marcescens* employs a “bet-hedging” strategy, diversifying into phenotypically distinct high- and low-virulence subpopulations to ensure population survival [109]. The dialogue extends to a complex molecular network where host-derived inflammatory signals (e.g., fatty acids) are metabolized by commensals like *Blautia producta* into derivatives that subsequently act as an “immune brake” to suppress excessive host inflammation, illustrating a feedback loop of mutual regulation [110]. This intricate three-way crosstalk is a central focus in models like *Clostridioides difficile* infection, where defining these interactions is key to understanding infection outcomes.

Therefore, the future of microbiota-based therapeutics lies in transitioning from a focus on “keystone species” to targeting keystone functions and ecological dynamics. This ecosystem-informed perspective suggests that interventions should aim to restore or stabilize the functional network of the community. Promising strategies include next-generation prebiotics designed to selectively nourish protective cross-feeding guilds, precision editing tools for pathogen-specific removal, and host pathway modulators that enhance innate mechanisms of microbial management. The integration of multi-omics data, computational ecology, and advanced ex vivo models will be essential to decipher these complex interactions and translate ecological principles into safe, effective, and personalized anti-infective therapies.

### 4.3. Core Defense Mechanisms and Therapeutic Translation

Synthesizing the myriad pathogen-specific interactions discussed herein reveals a set of convergent, core host defense modules orchestrated by the gut microbiota. We propose that these interconnected modules form the bedrock of colonization resistance:

The metabolite signaling network: A restricted set of microbiota-derived metabolites functions as critical host regulators. SCFAs like butyrate and acetate reinforce the epithelial barrier and modulate immune cell function via GPCR signaling and HDAC inhibition [32,33,83]. Tryptophan derivatives such as IPA signal through receptors like the AhR to regulate inflammation and directly inhibit some pathogens [76,90]. Secondary bile acids inhibit pathogens (e.g., *C. difficile*) and resolve inflammation via receptors like TGR5 [16,88]. Critically, these metabolites regulate host physiology not only through receptor binding but also via direct modulation of post-translational modifications, allowing rapid reshaping of the host proteome and epigenome in response to threats [111].

The systemic immune priming module: The microbiota is essential for educating and toning the host immune system, establishing a state of preparedness. This is exemplified by the gut–lung axis, where microbial metabolites (SCFAs, IPA) enhance alveolar macrophage function [82], optimize antiviral CD8^+^ T cell responses [85,87], and strengthen type I interferon production [83]. The recently elucidated APOL9–outer membrane vesicle (OMV) pathway represents a specialized, local instantiation of this module, wherein the host orchestrates a dialogue with *Bacteroidales* to prime the mucosal immune landscape [108]. It is necessary to clarify that the evidence and mechanisms about the gut–lung axis elaborated in this review are derived entirely from studies on respiratory infections, which may differ from those of other axes.

The mechanistic insights underlying these core modules are summarized in Table S1, which details key microbial strains, metabolites, and pathways identified in animal models of infection.

Therapeutic innovation is progressing toward more rational, mechanism-driven design of next-generation interventions, based on a deepening understanding of host–microbiota interactions.

### 4.4. The Dual Role of the Gut Microbiota in Infection

While this review has detailed the profound protective capacities of a healthy gut microbiota, the microbial community or its components can exacerbate or precipitate infectious disease under specific conditions. A complete view of the gut microbiota in infection must account for its capacity to both resist and facilitate disease. This duality highlights that microbial influence is not uniformly protective but is contingent on the specific interplay between the host, the resident microbes, and the invading pathogen.

In some cases, the microbiota directly aids pathogens. Certain enteric bacteria express molecules like histo-blood group antigens, which act as essential cofactors for viruses such as human norovirus, making bacterial presence a prerequisite for infection [112]. Similarly, respiratory viral infections can induce a dysbiotic state that depletes protective commensals and allows pathobionts like *Streptococcus pneumoniae* to expand, setting the stage for severe secondary bacterial pneumonia. This process is supported by studies showing that respiratory viruses can alter the gut microbiota, which in turn impairs the lung’s immune defenses against secondary bacterial infection, a key mechanism of the gut–lung axis in severe disease outcomes [113,114].

The effects of microbial metabolites are also context-dependent. SCFAs are traditionally celebrated for their barrier-strengthening and anti-inflammatory properties. However, their role as HDAC inhibitors can, in specific contexts, lead to the suppression of host antimicrobial gene programs. For instance, butyrate has been shown to enhance macrophage antimicrobial function against *Salmonella* [33], yet in models of respiratory viral infection followed by bacterial superinfection, it can paradoxically suppress protective type I interferon responses and downregulate key interferon-stimulated genes [115]. Conversely, in models of Japanese encephalitis virus infection, butyrate treatment was found to exacerbate disease progression by increasing viral load and neural cell death, potentially through the upregulation of pro-inflammatory cytokines and NF-κB signaling rather than interferon suppression [116]. Similarly, the tryptophan metabolite IAA demonstrates this duality. While involved in homeostasis, exogenous IAA administration has been found to exacerbate lung injury in murine bacterial pneumonia models, an effect mediated through the AhR that modulates immune cell responses, leading to worsened outcomes [91]. These examples underscore that metabolites are not universally “beneficial” or “detrimental.” Instead, they function as versatile components of host–microbe dialogue, with their net impact on infection being determined by the specific pathological and immunological landscape in which they act.

## 5. Conclusions

The gut microbiota constitutes a fundamental component of the host’s defense repertoire against infectious diseases. Its protective effects are mediated through an interwoven set of mechanisms, including direct pathogen inhibition, niche competition, barrier reinforcement, and systemic immunomodulation. Key microbial metabolites, such as short-chain fatty acids, secondary bile acids, and tryptophan derivatives, serve as critical signaling molecules in these processes. However, it is imperative to recognize that the microbiota functions not as a mere collection of beneficial species but as a complex adaptive system, wherein colonization resistance emerges from community-level interactions and a dynamic dialogue with the host.

Consequently, the future of microbiota-based therapeutics requires a paradigm shift—from a focus on individual microbial strains toward strategies that restore or stabilize the functional architecture of the microbial community. The rational design of next-generation interventions will depend on the convergence of multi-omics, computational modeling, and clinically informed experimentation. Ultimately, the goal is to develop personalized, microbiota-directed therapies that can complement conventional antimicrobials, offering a more nuanced and sustainable approach to mitigating the global burden of infectious diseases.

## Data Availability

Not applicable.

## Supplementary Materials

The following supporting information can be downloaded at: https://www.mdpi.com/article/10.3390/biology15030256/s1, Table S1: Summary of animal model studies on gut microbiota and infectious diseases [16,40,41,42,43,44,45,48,49,50,53,55,60,62,63,64,65,66,67,73,75,78,79,82,83,85,86,87,88,89,91,93,95].

## References

[1] Vonasek B.J., Radtke K.K., Vaz P., Buck W.C., Chabala C., McCollum E.D., Marcy O., Fitzgerald E., Kondwani A., Garcia-Prats A.J. (2022). Tuberculosis in children with severe acute malnutrition. Expert. Rev. Respir. Med..

[2] Majowicz S.E., Musto J., Scallan E., Angulo F.J., Kirk M., O’Brien S.J., Jones T.F., Fazil A., Hoekstra R.M., for the International Collaboration on Enteric Disease “Burden of Illness” Studies (2010). The Global Burden of Nontyphoidal *Salmonella Gastroenteritis*. Clin. Infect. Dis..

[3] Krammer F., Smith G.J.D., Fouchier R.A.M., Peiris M., Kedzierska K., Doherty P.C., Palese P., Shaw M.L., Treanor J., Webster R.G. (2018). Influenza. Nat. Rev. Dis. Primers.

[4] Hu B., Guo H., Zhou P., Shi Z.L. (2021). Characteristics of SARS-CoV-2 and COVID-19. Nat. Rev. Microbiol..

[5] Savage D.C. (1977). Microbial ecology of the gastrointestinal tract. Annu. Rev. Microbiol..

[6] Qin J., Li R., Raes J., Arumugam M., Burgdorf K.S., Manichanh C., Nielsen T., Pons N., Levenez F., Yamada T. (2010). A human gut microbial gene catalogue established by metagenomic sequencing. Nature.

[7] Kommineni S., Bretl D.J., Lam V., Chakraborty R., Hayward M., Simpson P., Cao Y., Bousounis P., Kristich C.J., Salzman N.H. (2015). Bacteriocin production augments niche competition by enterococci in the mammalian gastrointestinal tract. Nature.

[8] Corr S.C., Li Y., Riedel C.U., O’Toole P.W., Hill C., Gahan C.G. (2007). Bacteriocin production as a mechanism for the antiinfective activity of *Lactobacillus salivarius* UCC118. Proc. Natl. Acad. Sci. USA.

[9] Piewngam P., Zheng Y., Nguyen T.H., Dickey S.W., Joo H.-S., Villaruz A.E., Glose K.A., Fisher E.L., Hunt R.L., Li B. (2018). Pathogen elimination by probiotic *Bacillus* via signalling interference. Nature.

[10] Lay C.L., Dridi L., Bergeron M.G., Ouellette M., Fliss I.L. (2016). Nisin is an effective inhibitor of *Clostridium difficile* vegetative cells and spore germination. J. Med. Microbiol..

[11] Valenta C., Bernkop-Schnürch A., Rigler H.P. (1996). The antistaphylococcal effect of nisin in a suitable vehicle: A potential therapy for atopic dermatitis in man. J. Pharm. Pharmacol..

[12] Shin J.M., Gwak J.W., Kamarajan P., Fenno J.C., Rickard A.H., Kapila Y.L. (2016). Biomedical applications of nisin. J. Appl. Microbiol..

[13] Deriu E., Liu J.Z., Pezeshki M., Edwards R.A., Ochoa R.J., Contreras H., Libby S.J., Fang F.C., Raffatellu M. (2013). Probiotic bacteria reduce *Salmonella* typhimurium intestinal colonization by competing for iron. Cell Host Microbe.

[14] Momose Y., Hirayama K., Itoh K. (2008). Competition for proline between indigenous *Escherichia coli* and *E. coli* O157:H7 in gnotobiotic mice associated with infant intestinal microbiota and its contribution to the colonization resistance against *E. coli* O157:H7. Antonie Van Leeuwenhoek.

[15] Heinken A., Ravcheev D.A., Baldini F., Heirendt L., Fleming R.M.T., Thiele I. (2019). Systematic assessment of secondary bile acid metabolism in gut microbes reveals distinct metabolic capabilities in inflammatory bowel disease. Microbiome.

[16] Thanissery R., Winston J.A., Theriot C.M. (2017). Inhibition of spore germination, growth, and toxin activity of clinically relevant *C.difficile* strains by gut microbiota derived secondary bile acids. Anaerobe.

[17] Wexler A.G., Goodman A.L. (2017). An insider’s perspective: Bacteroides as a window into the microbiome. Nat. Microbiol..

[18] Turner J.R. (2009). Intestinal mucosal barrier function in health and disease. Nat. Rev. Immunol..

[19] Derrien M., Belzer C., de Vos W.M. (2017). *Akkermansia muciniphila* and its role in regulating host functions. Microb. Pathog..

[20] Khan S., Waliullah S., Godfrey V., Khan M.A.W., Ramachandran R.A., Cantarel B.L., Behrendt C., Peng L., Hooper L.V., Zaki H. (2020). Dietary simple sugars alter microbial ecology in the gut and promote colitis in mice. Sci. Transl. Med..

[21] Schroeder B.O., Birchenough G.M.H., Ståhlman M., Arike L., Johansson M.E.V., Hansson G.C., Bäckhed F. (2018). Bifidobacteria or Fiber Protects against Diet-Induced Microbiota-Mediated Colonic Mucus Deterioration. Cell Host Microbe.

[22] Topping D.L., Clifton P.M. (2001). Short-chain fatty acids and human colonic function: Roles of resistant starch and nonstarch polysaccharides. Physiol. Rev..

[23] Donohoe D.R., Garge N., Zhang X., Sun W., O’Connell T.M., Bunger M.K., Bultman S.J. (2011). The microbiome and butyrate regulate energy metabolism and autophagy in the mammalian colon. Cell Metab..

[24] Kelly C.J., Zheng L., Campbell E.L., Saeedi B., Scholz C.C., Bayless A.J., Wilson K.E., Glover L.E., Kominsky D.J., Magnuson A. (2015). Crosstalk between Microbiota-Derived Short-Chain Fatty Acids and Intestinal Epithelial HIF Augments Tissue Barrier Function. Cell Host Microbe.

[25] Singh R., Chandrashekharappa S., Bodduluri S.R., Baby B.V., Hegde B., Kotla N.G., Hiwale A.A., Saiyed T., Patel P., Vijay-Kumar M. (2019). Enhancement of the gut barrier integrity by a microbial metabolite through the Nrf2 pathway. Nat. Commun..

[26] Lundell A.C., Bjornsson V., Ljung A., Ceder M., Johansen S., Lindhagen G., Tornhage C.J., Adlerberth I., Wold A.E., Rudin A. (2012). Infant B cell memory differentiation and early gut bacterial colonization. J. Immunol..

[27] Rhayat L., Maresca M., Nicoletti C., Perrier J., Brinch K.S., Christian S., Devillard E., Eckhardt E. (2019). Effect of *Bacillus subtilis* Strains on Intestinal Barrier Function and Inflammatory Response. Front. Immunol..

[28] Gebremariam H.G., Qazi K.R., Somiah T., Pathak S.K., Sjölinder H., Sverremark Ekström E., Jonsson A.B. (2019). *Lactobacillus gasseri* Suppresses the Production of Proinflammatory Cytokines in *Helicobacter pylori*-Infected Macrophages by Inhibiting the Expression of ADAM17. Front. Immunol..

[29] Kamada N., Seo S.U., Chen G.Y., Nunez G. (2013). Role of the gut microbiota in immunity and inflammatory disease. Nat. Rev. Immunol..

[30] Guo W., Xiang Q., Mao B., Tang X., Cui S., Li X., Zhao J., Zhang H., Chen W. (2021). Protective Effects of Microbiome-Derived Inosine on Lipopolysaccharide-Induced Acute Liver Damage and Inflammation in Mice via Mediating the TLR4/NF-κB Pathway. J. Agric. Food Chem..

[31] Li B., Zhang H., Shi L., Li R., Luo Y., Deng Y., Li S., Li R., Liu Z. (2022). *Saccharomyces boulardii* alleviates DSS-induced intestinal barrier dysfunction and inflammation in humanized mice. Food Funct..

[32] Singh N., Gurav A., Sivaprakasam S., Brady E., Padia R., Shi H., Thangaraju M., Prasad P.D., Manicassamy S., Munn D.H. (2014). Activation of Gpr109a, receptor for niacin and the commensal metabolite butyrate, suppresses colonic inflammation and carcinogenesis. Immunity.

[33] Schulthess J., Pandey S., Capitani M., Rue-Albrecht K.C., Arnold I., Franchini F., Chomka A., Ilott N.E., Johnston D.G.W., Pires E. (2019). The Short Chain Fatty Acid Butyrate Imprints an Antimicrobial Program in Macrophages. Immunity.

[34] Agus A., Planchais J., Sokol H. (2018). Gut microbiota regulation of tryptophan metabolism in health and disease. Cell Host Microbe.

[35] Scott S.A., Fu J., Chang P.V. (2024). Dopamine receptor D2 confers colonization resistance via microbial metabolites. Nature.

[36] Hagihara M., Yamashita M., Ariyoshi T., Eguchi S., Minemura A., Miura D., Higashi S., Oka K., Nonogaki T., Mori T. (2022). Clostridium butyricum-induced ω-3 fatty acid 18-HEPE elicits anti-influenza virus pneumonia effects through interferon-λ upregulation. Cell Rep..

[37] Cait A., Hughes M.R., Antignano F., Cait J., Dimitriu P.A., Maas K.R., Reynolds L.A., Hacker L., Mohr J., Finlay B.B. (2018). Microbiome-driven allergic lung inflammation is ameliorated by short-chain fatty acids. Mucosal Immunol..

[38] d’Ettorre G., Ceccarelli G., Marazzato M., Campagna G., Pinacchio C., Alessandri F., Ruberto F., Rossi G., Celani L., Scagnolari C. (2020). Challenges in the Management of SARS-CoV2 Infection: The Role of Oral Bacteriotherapy as Complementary Therapeutic Strategy to Avoid the Progression of COVID-19. Front Med.

[39] Ma B., Mei X., Lei C., Li C., Gao Y., Kong L., Zhai X., Wang H. (2020). Enrofloxacin Shifts Intestinal Microbiota and Metabolic Profiling and Hinders Recovery from *Salmonella enterica* subsp. *enterica* Serovar Typhimurium Infection in Neonatal Chickens. mSphere.

[40] Strempel A., Weiss A.S., Wittmann J., Salvado Silva M., Ring D., Wortmann E., Clavel T., Debarbieux L., Kleigrewe K., Stecher B. (2023). Bacteriophages targeting protective commensals impair resistance against *Salmonella* Typhimurium infection in gnotobiotic mice. PLoS Pathog..

[41] Cherrak Y., Younes A.A., Perez-Molphe-Montoya E., Maurer L., Yilmaz K., Enz U., Zeder C., Kiefer P., Christen P., Gül E. (2025). Neutrophil recruitment during intestinal inflammation primes *Salmonella* elimination by commensal *E. coli* in a context-dependent manner. Cell Host Microbe.

[42] Liu J., Liu H., Liu H., Teng Y., Qin N., Ren X., Xia X. (2023). Live and pasteurized *Akkermansia muciniphila* decrease susceptibility to *Salmonella* Typhimurium infection in mice. J. Adv. Res..

[43] Beresford-Jones B.S., Suyama S., Clare S., Soderholm A., Xia W., Sardar P., Lee J., Harcourt K., Lawley T.D., Pedicord V.A. (2025). *Enterocloster clostridioformis* protects against Salmonella pathogenesis and modulates epithelial and mucosal immune function. Microbiome.

[44] Li H., Ma X., Shang Z., Liu X., Qiao J. (2024). Lactobacillus acidophilus alleviate Salmonella enterica Serovar Typhimurium-induced murine inflammatory/oxidative responses via the p62-Keap1-Nrf2 signaling pathway and cecal microbiota. Front. Microbiol..

[45] Kong Q., Shang Z., Liu Y., Fakhar E.A.K.M., Suo-Lang S., Xu Y., Tan Z., Li J., Liu S. (2022). Preventive effect of *Terminalia bellirica* (Gaertn.) Roxb. extract on mice infected with *Salmonella Typhimurium*. Front. Cell Infect. Microbiol..

[46] Pickard J.M., Porwollik S., Caballero-Flores G., Caruso R., Fukuda S., Soga T., Inohara N., McClelland M., Núñez G. (2025). Dietary amino acids regulate *Salmonella* colonization via microbiota-dependent mechanisms in the mouse gut. Nat. Commun..

[47] Yang Z., Lin Z., You Y., Zhang M., Gao N., Wang X., Peng J., Wei H. (2025). Gut Microbiota-Derived Hyocholic Acid Enhances Type 3 Immunity and Protects Against *Salmonella enterica* Serovar Typhimurium in Neonatal Rats. Adv. Sci..

[48] Azcarate-Peril M.A., Butz N., Cadenas M.B., Koci M., Ballou A., Mendoza M., Ali R., Hassan H. (2018). An Attenuated *Salmonella enterica* Serovar Typhimurium Strain and Galacto-Oligosaccharides Accelerate Clearance of *Salmonella* Infections in Poultry through Modifications to the Gut Microbiome. Appl. Environ. Microbiol..

[49] Cui X., Zhang S., Jiang S., Gou Z., Wang Y. (2023). Dietary protocatechuic acid ameliorates ileal mucosal barrier injury and inflammatory response and improves intestinal microbiota composition in Yellow chickens challenged with *Salmonella typhimurium*. Poult. Sci..

[50] Jacobson A., Lam L., Rajendram M., Tamburini F., Honeycutt J., Pham T., Van Treuren W., Pruss K., Stabler S.R., Lugo K. (2018). A Gut Commensal-Produced Metabolite Mediates Colonization Resistance to *Salmonella* Infection. Cell Host Microbe.

[51] Wang Z., Kang S., Wu Z., Liu X., Zhang X., Wu Y., Wen Y., Zhou X., Zhang G., Wang J. (2025). *Muribaculum intestinale* restricts *Salmonella* Typhimurium colonization by converting succinate to propionate. Isme J..

[52] Shelton C.D., Yoo W., Shealy N.G., Torres T.P., Zieba J.K., Calcutt M.W., Foegeding N.J., Kim D., Kim J., Ryu S. (2022). *Salmonella enterica* serovar Typhimurium uses anaerobic respiration to overcome propionate-mediated colonization resistance. Cell Rep..

[53] Xu W., Bo J., Jia L., Zhu K., Luo Q. (2025). Melatonin Confers Protection Against Multidrug-Resistant Bacterial Infections in Aged Mice Via Microbiota-Derived Butyrate. J. Pineal Res..

[54] Schubert C., Näf J., Petukhov L., Laganenka L., Cherrak Y., Hardt W.D. (2025). Strain-specific galactose utilization by commensal *E. coli* mitigates *Salmonella* establishment in the gut. PLoS Pathog..

[55] Alavi S., Mitchell J.D., Cho J.Y., Liu R., Macbeth J.C., Hsiao A. (2020). Interpersonal Gut Microbiome Variation Drives Susceptibility and Resistance to Cholera Infection. Cell.

[56] Barrasso K., Chac D., Debela M.D., Geigel C., Steenhaut A., Rivera Seda A., Dunmire C.N., Harris J.B., Larocque R.C., Midani F.S. (2022). Impact of a human gut microbe on Vibrio cholerae host colonization through biofilm enhancement. Elife.

[57] Yoon M.Y., Min K.B., Lee K.M., Yoon Y., Kim Y., Oh Y.T., Lee K., Chun J., Kim B.Y., Yoon S.H. (2016). A single gene of a commensal microbe affects host susceptibility to enteric infection. Nat. Commun..

[58] Chen J., Byun H., Liu R., Jung I.J., Pu Q., Zhu C.Y., Tanchoco E., Alavi S., Degnan P.H., Ma A.T. (2022). A commensal-encoded genotoxin drives restriction of *Vibrio cholerae* colonization and host gut microbiome remodeling. Proc. Natl. Acad. Sci. USA.

[59] Bachmann V., Kostiuk B., Unterweger D., Diaz-Satizabal L., Ogg S., Pukatzki S. (2015). Bile Salts Modulate the Mucin-Activated Type VI Secretion System of Pandemic *Vibrio cholerae*. PLoS Negl. Trop. Dis..

[60] Bosire E.M., Adams M.C., Pavinski Bitar P.D., Murphy S.G., Shin J.H., Chappie J.S., Dörr T., Altier C. (2025). *Vibrio cholerae* integrates interspecies quorum-sensing signals to regulate virulence. mBio.

[61] Gorelik O., Rogad A., Holoidovsky L., Meijler M.M., Sal-Man N. (2022). Indole intercepts the communication between enteropathogenic *E. coli* and *Vibrio cholerae*. Gut Microbes.

[62] You J.S., Yong J.H., Kim G.H., Moon S., Nam K.T., Ryu J.H., Yoon M.Y., Yoon S.S. (2019). Commensal-derived metabolites govern Vibrio cholerae pathogenesis in host intestine. Microbiome.

[63] Patra S., Pradhan B., Roychowdhury A. (2025). Complete genome sequence, metabolic profiling and functional studies reveal *Ligilactobacillus salivarius* LS-ARS2 is a promising biofilm-forming probiotic with significant antioxidant, antibacterial, and antibiofilm potential. Front. Microbiol..

[64] Di Luccia B., Ahern P.P., Griffin N.W., Cheng J., Guruge J.L., Byrne A.E., Rodionov D.A., Leyn S.A., Osterman A.L., Ahmed T. (2020). Combined Prebiotic and Microbial Intervention Improves Oral Cholera Vaccination Responses in a Mouse Model of Childhood Undernutrition. Cell Host Microbe.

[65] Tian S., Kim M.S., Zhao J., Heber K., Hao F., Koslicki D., Tian S., Singh V., Patterson A.D., Bisanz J.E. (2025). A designed synthetic microbiota provides insight to community function in *Clostridioides difficile* resistance. Cell Host Microbe.

[66] Li X., Wu X., Zang W., Zhou Z., Cui W., Chen Y., Yang H. (2025). Dietary iron attenuates *Clostridioides difficile* infection via modulation of intestinal immune response and gut microbiota. Virulence.

[67] Yong C.C., Lim J., Kim B.K., Park D.J., Oh S. (2019). Suppressive effect of Lactobacillus fermentum Lim2 on *Clostridioides difficile* 027 toxin production. Lett. Appl. Microbiol..

[68] Darkoh C., Plants-Paris K., Bishoff D., DuPont H.L. (2019). *Clostridium difficile* Modulates the Gut Microbiota by Inducing the Production of Indole, an Interkingdom Signaling and Antimicrobial Molecule. mSystems.

[69] Gaisawat M.B., MacPherson C.W., Tremblay J., Piano A., Iskandar M.M., Tompkins T.A., Kubow S. (2019). Probiotic Supplementation in a *Clostridium difficile*-Infected Gastrointestinal Model Is Associated with Restoring Metabolic Function of Microbiota. Microorganisms.

[70] Wipperman M.F., Fitzgerald D.W., Juste M.A.J., Taur Y., Namasivayam S., Sher A., Bean J.M., Bucci V., Glickman M.S. (2017). Antibiotic treatment for Tuberculosis induces a profound dysbiosis of the microbiome that persists long after therapy is completed. Sci. Rep..

[71] Namasivayam S., Maiga M., Yuan W., Thovarai V., Costa D.L., Mittereder L.R., Wipperman M.F., Glickman M.S., Dzutsev A., Trinchieri G. (2017). Longitudinal profiling reveals a persistent intestinal dysbiosis triggered by conventional anti-tuberculosis therapy. Microbiome.

[72] Dumas A., Corral D., Colom A., Levillain F., Peixoto A., Hudrisier D., Poquet Y., Neyrolles O. (2018). The Host Microbiota Contributes to Early Protection Against Lung Colonization by *Mycobacterium tuberculosis*. Front. Immunol..

[73] Negi S., Pahari S., Bashir H., Agrewala J.N. (2019). Gut Microbiota Regulates Mincle Mediated Activation of Lung Dendritic Cells to Protect Against *Mycobacterium tuberculosis*. Front. Immunol..

[74] Yang F., Yang Y., Chen Y., Li G., Zhang G., Chen L., Zhang Z., Mai Q., Zeng G. (2020). MiR-21 Is Remotely Governed by the Commensal Bacteria and Impairs Anti-TB Immunity by Down-Regulating IFN-γ. Front. Microbiol..

[75] Yang F., Yang Y., Chen L., Zhang Z., Liu L., Zhang C., Mai Q., Chen Y., Chen Z., Lin T. (2022). The gut microbiota mediates protective immunity against tuberculosis via modulation of lncRNA. Gut Microbes.

[76] Negatu D.A., Yamada Y., Xi Y., Go M.L., Zimmerman M., Ganapathy U., Dartois V., Gengenbacher M., Dick T. (2019). Gut Microbiota Metabolite Indole Propionic Acid Targets Tryptophan Biosynthesis in *Mycobacterium tuberculosis*. mBio.

[77] Baral T., Johnson A.S., Unnikrishnan M.K., Manu M.K., Saravu K., Udyavara Kudru C., Abdulsalim S., Singh J., Mukhopadhyay C., Rao M. (2025). Potential role of indole-3-propionic acid in tuberculosis: Current perspectives and future prospects. Expert. Opin. Ther. Targets.

[78] Yu Z., Shen X., Wang A., Hu C., Chen J. (2023). The gut microbiome: A line of defense against tuberculosis development. Front. Cell Infect. Microbiol..

[79] Hu X., Sun X., Zhao Y., Iv C., Sun X., Jin M., Zhang Q. (2023). GlcNac produced by the gut microbiome enhances host influenza resistance by modulating NK cells. Gut Microbes.

[80] Wu X., Li R.F., Lin Z.S., Xiao C., Liu B., Mai K.L., Zhou H.X., Zeng D.Y., Cheng S., Weng Y.C. (2023). Coinfection with influenza virus and non-typeable *Haemophilus influenzae* aggregates inflammatory lung injury and alters gut microbiota in COPD mice. Front. Microbiol..

[81] Hu X., Zhao Y., Yang Y., Gong W., Sun X., Yang L., Zhang Q., Jin M. (2020). Akkermansia muciniphila Improves Host Defense Against Influenza Virus Infection. Front. Microbiol..

[82] Ngo V.L., Lieber C.M., Kang H.J., Sakamoto K., Kuczma M., Plemper R.K., Gewirtz A.T. (2024). Intestinal microbiota programming of alveolar macrophages influences severity of respiratory viral infection. Cell Host Microbe.

[83] Niu J., Cui M., Yang X., Li J., Yao Y., Guo Q., Lu A., Qi X., Zhou D., Zhang C. (2023). Microbiota-derived acetate enhances host antiviral response via NLRP3. Nat. Commun..

[84] Hornung F., SureshKumar H.K., Klement L., Reisser Y., Wernike C., Nischang V., Jordan P.M., Werz O., Hoffmann C., Löffler B. (2025). High-fat diet impairs microbial metabolite production and aggravates influenza A infection. Cell Commun. Signal.

[85] Qiu J., Shi C., Zhang Y., Niu T., Chen S., Yang G., Zhu S.J., Wang C. (2024). Microbiota-derived acetate is associated with functionally optimal virus-specific CD8^+^ T cell responses to influenza virus infection via GPR43-dependent metabolic reprogramming. Gut Microbes.

[86] Saint-Martin V., Guillory V., Chollot M., Fleurot I., Kut E., Roesch F., Caballero I., Helloin E., Chambellon E., Ferguson B. (2024). The gut microbiota and its metabolite butyrate shape metabolism and antiviral immunity along the gut-lung axis in the chicken. Commun. Biol..

[87] Stevens J., Culberson E., Kinder J., Ramiriqui A., Gray J., Bonfield M., Shao T.Y., Al Gharaibeh F., Peterson L., Steinmeyer S. (2025). Microbiota-derived inosine programs protective CD8^+^ T cell responses against influenza in newborns. Cell.

[88] He S., Lu S., Yang T., Ma H., He Y., Mi J., Yue K., Huang Y., Song L., Xiao Y. (2025). *Bacteroides dorei* RX2020-derived bile acid alleviates influenza virus infection through TGR5 signaling. Cell Commun. Signal.

[89] Mao Z., Liu C., Ni J., Huang M., Qu W., Chen W., Shen Y., Qin T., Gao M., Zheng S. (2025). Gut derived (S)-Equol mitigates influenza viral pneumonia by modulating macrophage polarization via Nrf2 mediated AKT/ERK/NF-κb signaling pathways. Free Radic. Biol. Med..

[90] Heumel S., de Rezende Rodovalho V., Urien C., Specque F., Brito Rodrigues P., Robil C., Delval L., Sencio V., Descat A., Deruyter L. (2024). Shotgun metagenomics and systemic targeted metabolomics highlight indole-3-propionic acid as a protective gut microbial metabolite against influenza infection. Gut Microbes.

[91] Kullberg R.F.J., van Linge C.C.A., Haak B.W., Paul P.S., Butler J.M., Wolff N., van Engelen T.S.R., Sikkens J.J., Bomers M.K., Lefèvre A. (2025). Effect of the gut microbiota-derived tryptophan metabolite indole-3-acetic acid in pneumonia. Nat. Commun..

[92] Martino C., Kellman B.P., Sandoval D.R., Clausen T.M., Cooper R., Benjdia A., Soualmia F., Clark A.E., Garretson A.F., Marotz C.A. (2025). SARS-CoV-2 infectivity can be modulated through bacterial grooming of the glycocalyx. mBio.

[93] Brown J.A., Sanidad K.Z., Lucotti S., Lieber C.M., Cox R.M., Ananthanarayanan A., Basu S., Chen J., Shan M., Amir M. (2022). Gut microbiota-derived metabolites confer protection against SARS-CoV-2 infection. Gut Microbes.

[94] Song I., Yang J., Saito M., Hartanto T., Nakayama Y., Ichinohe T., Fukuda S. (2024). Prebiotic inulin ameliorates SARS-CoV-2 infection in hamsters by modulating the gut microbiome. NPJ Sci. Food.

[95] Schreiber S., Waetzig G.H., López-Agudelo V.A., Geisler C., Schlicht K., Franzenburg S., di Giuseppe R., Pape D., Bahmer T., Krawczak M. (2025). Nicotinamide modulates gut microbial metabolic potential and accelerates recovery in mild-to-moderate COVID-19. Nat. Metab..

[96] Walter J., Armet A.M., Finlay B.B., Shanahan F. (2020). Establishing or Exaggerating Causality for the Gut Microbiome: Lessons from Human Microbiota-Associated Rodents. Cell.

[97] Wang L., Chen X., Pollock N.R., Villafuerte Gálvez J.A., Alonso C.D., Wang D., Daugherty K., Xu H., Yao J., Chen Y. (2025). Metagenomic analysis reveals distinct patterns of gut microbiota features with diversified functions in *C. difficile* infection (CDI), asymptomatic carriage and non-CDI diarrhea. Gut Microbes.

[98] Wipperman M.F., Bhattarai S.K., Vorkas C.K., Maringati V.S., Taur Y., Mathurin L., McAulay K., Vilbrun S.C., Francois D., Bean J. (2021). Gastrointestinal microbiota composition predicts peripheral inflammatory state during treatment of human tuberculosis. Nat. Commun..

[99] Feuerstadt P., Louie T.J., Lashner B., Wang E.E.L., Diao L., Bryant J.A., Sims M., Kraft C.S., Cohen S.H., Berenson C.S. (2022). SER-109, an Oral Microbiome Therapy for Recurrent *Clostridioides difficile* Infection. N. Engl. J. Med..

[100] Louie T., Golan Y., Khanna S., Bobilev D., Erpelding N., Fratazzi C., Carini M., Menon R., Ruisi M., Norman J.M. (2023). VE303, a Defined Bacterial Consortium, for Prevention of Recurrent *Clostridioides difficile* Infection: A Randomized Clinical Trial. JAMA.

[101] Holthaus D., Kraft M.R., Krug S.M., Wolf S., Müller A., Delgado Betancourt E., Schorr M., Holland G., Knauf F., Schulzke J.-D. (2022). Dissection of Barrier Dysfunction in Organoid-Derived Human Intestinal Epithelia Induced by *Giardia duodenalis*. Gastroenterology.

[102] Hashimi M., Sebrell T.A., Hedges J.F., Snyder D., Lyon K.N., Byrum S.D., Mackintosh S.G., Crowley D., Cherne M.D., Skwarchuk D. (2023). Antiviral responses in a Jamaican fruit bat intestinal organoid model of SARS-CoV-2 infection. Nat. Commun..

[103] Mead B.E., Hattori K., Levy L., Imada S., Goto N., Vukovic M., Sze D., Kummerlowe C., Matute J.D., Duan J. (2022). Screening for modulators of the cellular composition of gut epithelia via organoid models of intestinal stem cell differentiation. Nat. Biomed. Eng..

[104] Lawley T.D., Walker A.W. (2013). Intestinal colonization resistance. Immunology.

[105] Kamada N., Chen G.Y., Inohara N., Núñez G. (2013). Control of pathogens and pathobionts by the gut microbiota. Nat. Immunol..

[106] Zhang M., Shi S., Feng Y., Zhang F., Xiao Y., Li X., Pan X., Feng Y., Liu D., Guo Y. (2025). Synthetic microbial community improves chicken intestinal homeostasis and provokes anti-*Salmonella* immunity mediated by segmented filamentous bacteria. ISME J..

[107] Zhou Z., Yang M., Fang H., Zhang B., Ma Y., Li Y., Liu Y., Cheng Z., Zhao Y., Si Z. (2025). Tailoring a Functional Synthetic Microbial Community Alleviates *Fusobacterium nucleatum*-infected Colorectal Cancer via Ecological Control. Adv. Sci..

[108] Yang T., Hu X., Cao F., Yun F., Jia K., Zhang M., Kong G., Nie B., Liu Y., Zhang H. (2025). Targeting symbionts by apolipoprotein L proteins modulates gut immunity. Nature.

[109] Zhang S., Wang Z., Liu A., Li J., Zhuang J., Ji X., Mulama P.I., Li M., Cao H., Tan E.K. (2025). Hosts and Commensal Bacteria Synergistically Antagonize Opportunistic Pathogens at the Single-Cell Resolution. Adv. Sci..

[110] Hou J., Song X. (2025). Inter-kingdom lipid messengers sustain gut harmony. Cell Host Microbe.

[111] Li S., Guo Y., An S., Ge L., You J., Ren W. (2025). Gut microbiota-host post-translational modification axis in immunometabolic diseases. Trends Immunol..

[112] Jones M.K., Watanabe M., Zhu S., Graves C.L., Keyes L.R., Grau K.R., Gonzalez-Hernandez M.B., Iovine N.M., Wobus C.E., Vinjé J. (2014). Enteric bacteria promote human and mouse norovirus infection of B cells. Science.

[113] Sencio V., Machado M.G., Trottein F. (2021). The lung-gut axis during viral respiratory infections: The impact of gut dysbiosis on secondary disease outcomes. Mucosal Immunol..

[114] Lenhard A., Joma B.H., Siwapornchai N., Hakansson A.P., Leong J.M., Bou Ghanem E.N. (2022). A Mouse Model for the Transition of *Streptococcus pneumoniae* from Colonizer to Pathogen upon Viral Co-Infection Recapitulates Age-Exacerbated Illness. J. Vis. Exp..

[115] Chemudupati M., Kenney A.D., Smith A.C., Fillinger R.J., Zhang L., Zani A., Liu S.L., Anderson M.Z., Sharma A., Yount J.S. (2020). Butyrate Reprograms Expression of Specific Interferon-Stimulated Genes. J. Virol..

[116] Satheesan A., Sharma S., Basu A. (2023). Sodium Butyrate Induced Neural Stem/Progenitor Cell Death in an Experimental Model of Japanese Encephalitis. Metab. Brain Dis..

