# Supplementary material for: Role and Mechanisms of Gut Microbiota in Infectious Diseases: Recent Evidence from Animal Models
Source: Biology (Basel). 2026 Jan 30;15(3):256. doi: 10.3390/biology15030256 (PMC12897338; doi:10.3390/biology15030256)
Supplement: Supplementary file 1 [file biology-15-00256-s001.zip › biology-4051833-supplementary.pdf]

Table S1. Summary of animal model studies on gut microbiota and infectious diseases.

Disease	Model	Bacteria /Metabolite	Mechanism	Reference	Year
***Salmonella* Typhimurium Infection**	Germ-free mouse model	Commensal *E. coli*	Recruit neutrophils and occupies ecological niches.	[40, 41]	2023, 2025
	Germ-free mouse model	*A. muciniphila*	Promotes NLRP3 inflammasome activation and enhances macrophage antimicrobial functions, reducing host susceptibility.	[42]	2023
	Germ-free mouse model	*E. clostridioformis*	Protects against *Salmonella* pathogenesis by enhancing epithelial barrier function and modulating mucosal immune responses.	[43]	2025
	Germ-free mice	*Lactobacillus acidophilus*	Activates the p62-Keap1-Nrf2 signaling pathway and modulates cecal microbiota composition, alleviating inflammation and oxidative stress.	[44]	2024
	Neonatal rat model	*S. boulardii,* Hyocholic Acid	Potentiates type 3 immunity, enhancing host defense against Salmonella.	[45]	2025
	Commercial broiler chick model	Galacto-oligosaccharides,	Modulates gut microbiota composition	[48]	2018
	Female yellow broiler model	Protocatechuic acid	Modulates gut microbiota composition	[49]	2023
	Mouse model	Propionate	Directly inhibits *S.* Typhimurium growth by disrupting intracellular pH homeostasis.	[50]	2018
	Aged mouse model	Butyrate	Enhances host defense through immunomodulatory effects.	[53]	2025
**Cholera**	Germ-free mouse model	*cis*-2-Decenoic acid	Binding directly to ToxT in *V. cholerae*, accelerating its degradation and inhibiting toxin expression.	[60]	2020
	In vitro co-culture model simulating small intestinal conditions	Indole	Disrupts interspecies communication between *V. cholerae* and enteropathogenic *E. coli*, influencing pathogen virulence.	[62]	2022
	Germ-free mouse model	SCFAs	Modulates the intestinal microenvironment, inhibiting *V. cholerae* colonization.	[63, 64]	2019, 2025
	Infant CD-1 mouse model	Bile Acids	Inhibit T6SS-mediated killing of commensal bacteria, enhancing intestinal immunity and protecting against *V. cholera* infection.	[55]	2015
***Clostridioides difficile* Infection**	Mouse model	Dietary iron	Modulates intestinal immune responses and enriches specific protective bacteria.	[66]	2025
	Germ-free mouse model	Strains performing stickland fermentation	Competitive nutrient utilization *via* Stickland fermentation pathway	[65]	2025
	In vitro experimental model	*Lactobacillus fermentum* Lim2	Inhibits *C. difficile* toxin production by disrupting its quorum-sensing system.	[67]	2019
	In vitro experimental model	SBAs	Inhibits *C. difficile* spore germination, growth, and toxin production.	[16]	2017

Tuberculosis	Mouse model	Lactobacillus	Restores mincle expression on lung dendritic cells and enhances anti-*Mycobacterium tuberculosis* response.	[73]	2019
	Mouse model	*Bacteroides fragilis*	Acts as a direct regulator promoting anti-TB immunity through lncRNA regulation.	[75]	2022
	Pregnant mouse model	SCFAs	Can be transported to the lungs and may remodel the lung microbiome and immunity *via* the "gut-lung axis" in TB.	[78]	2023
Influenza Infection	Mouse model	*Clostridium* spp., *Phocaeicola sartorii*, *A. muciniphila*	Upregulates intestinal N-acetyl-D-glucosamine (GlcNAc) levels, mediating anti-influenza effects by increasing the proportion and activity of NK cells.	[79]	2023
	Germ-free mouse model	Segmented filamentous bacteria	Enhances antiviral immunity by activating alveolar macrophages.	[82]	2024
	Nlrp3−/− mouse model / Germ-free mouse model	Acetate	Augments IFN-I production *via* the NLRP3-GPR43-MAVS signaling axis and optimizes virus-specific $CD8^+$ T cell responses.	[83, 85]	2023, 2024
	Chicken model	Butyrate	Regulates ISGs in chicken respiratory epithelial cells	[86]	2024
	Mouse model	Inosine	Crucial for programming protective $CD8^+$ T cell immunity against influenza in early life by enabling NFIL3-dependent epigenetic regulation of TCF1.	[87]	2025
	Mouse model	Secondary Bile Acids	Activates the lung TGR5-cAMP-PKA pathway to suppress inflammation during infection.	[88]	2025
	Mouse model	(S)-Equol	Alleviates viral pneumonia by activating the transcription factor Nrf2 in macrophages, inhibiting pro-inflammatory AKT/ERK/NF-κB signaling.	[89]	2025
	Mouse model	IPA	Mitigates viral load and pulmonary inflammation.	[91]	2024
COVID-19	Mouse/hamster model	SCFAs	Downregulates ACE2 expression and activates GPR41/GPR43 involved in enhancing antiviral immunity, mitigating viral entry and load.	[93]	2022
	Syrian hamster model	Nicotinamide	modulate tryptophan metabolism	[95]	2025

Unless otherwise specified, all animal models used were specific pathogen-free (SPF) grade.
